# Global RECHARGE: Establishing a standard international data set for pulmonary rehabilitation in low- and middle-income countries

**DOI:** 10.7189/jogh.10.020316

**Published:** 2020-12

**Authors:** Mark W Orme, Robert C Free, Adrian Manise, Amy V Jones, Azamat Akylbekov, Andy Barton, Berik Emilov, Bhushan Girase, Akila R Jayamaha, Rupert Jones, Winceslaus Katagira, Bruce Kirenga, Jesse Matheson, Ruhme Miah, Chamilya Perrera, Shruti Sahasrabudhe, Sundeep Salvi, Rogers Sekibira, Talant Sooronbaev, Michael C Steiner, Savi Wimalasekera, Sally J Singh

**Affiliations:** 1 Respiratory Sciences, University of Leicester, Leicester, UK; 2 Centre for Exercise and Rehabilitation Science, NIHR Leicester Biomedical Research Centre-Respiratory, Leicester, UK; 3 NIHR Leicester Biomedical Research Centre, Leicester, UK; 4 Department of Respiratory, National Centre of Cardiology and Internal Medicine, Bishkek, Kyrgyzstan; 5 Faculty of Health, University of Plymouth, Plymouth, UK; 6 Chest Research Foundation, Pune, India; 7 Department of Health Sciences, Kaatsu International University, Battaramulla, Sri Lanka; 8 Makerere University Lung Institute, Makerere University College of Health Sciences, Mulago Hospital, Kampala, Uganda; 9 Department of Economics, University of Sheffield, Sheffield, UK; 10 Department of Physiology, Faculty of Medical Sciences, University of Sri Jayewardenepura, Nugegoda, Sri Lanka

Chronic respiratory diseases (CRD) are highly prevalent in low- and middle-income countries (LMICs). People living with CRD are often disabled by breathlessness which can result in reduced health-related quality of life, including reduced exercise tolerance, significant psychological morbidity and reduced ability to work. Implementing clinically and cost-effective interventions to tackle these problems can be challenging in low-resource settings. Pulmonary rehabilitation is a low cost, high impact intervention that reverses CRD-related disability and is supported by the highest level of research. Pulmonary rehabilitation is delivered by a multidisciplinary team and has exercise training and education at its core to support effective disease management and improve people’s quality of life. There is an unmet need for pulmonary rehabilitation that is profound in LMICs where the demand greatly outweighs the capacity. The sparse existence of pulmonary rehabilitation in LMICs offers an important opportunity to support the expansion of high quality, benchmarked services as it becomes increasingly recognised and available. Quality assurance procedures for pulmonary rehabilitation in the developed world are now in place; helping to ensure a high standard of patient care. In this paper we discuss a common data set that has been developed by the NIHR Global Health Research Group on Respiratory Rehabilitation (Global RECHARGE). Standardising data collection with a pre-determined set of measurements is proposed whereby collaborators will use common data collection tools and procedures. Benchmarking and quality improvement with continuous audit offer a potential to maximise benefits, reduce waste and improve patient outcomes. We welcome expressions of interest from health care professionals and researchers from LMICs, including groups looking to strengthen their local research capacity and from those looking to set up pulmonary rehabilitation through to those already running a service. We believe the wide adoption of this core data set will facilitate quality assurance of pulmonary rehabilitation programmes, provide opportunities to expand services over time, de novo research opportunities offered by trans-national data and enhanced research capacity in partner organisations.

## GLOBAL BURDEN OF RESPIRATORY DISEASE

Chronic respiratory diseases (CRD) have a high prevalence across the developing world, with more than 90% of deaths occurring in low- and middle- income countries (LMICs) [1]. Estimated global chronic obstructive pulmonary disease (COPD) prevalence is 10.7% [2]. People living with CRD are often disabled by breathlessness and may result in reduced exercise capacity, poor quality of life, social isolation, significant psychological morbidity and reduced ability to work [3]. For individuals with CRD, these problems may be exacerbated by socio-economic disadvantage or geographical isolation [4]. Implementing clinically and cost-effective interventions to ameliorate these problems is a clear public health priority but can be challenging in low-resource settings.

## PULMONARY REHABILITATION

Pulmonary rehabilitation is a low cost, high impact intervention that reverses the disability associated with CRDs, is supported by the highest level of research evidence and is recommended in all national and international guidelines [5,6]. Pulmonary rehabilitation is delivered by a multidisciplinary team and consists of exercise training and education to support effective disease management, improve exercise capacity and reduce symptom burden to allow individuals to be economically productive and fulfil meaningful roles [7]. The WHO Rehabilitation 2030: Call to Action makes the case for the fundamental role of accessible and affordable rehabilitation and acknowledges an unmet need that is profound in LMICs [7] where the demand for pulmonary rehabilitation greatly outweighs the capacity [8].

## AN INTERNATIONAL DATA SET OF COMMON MEASURES FOR PULMONARY REHABILITATION TRIALS ACROSS LMICS

The sparse existence of pulmonary rehabilitation in LMICs offers an important opportunity to support the expansion of high quality, benchmarked pulmonary rehabilitation services in LMICs as this service becomes increasingly recognised and available. There is evidence to suggest pulmonary rehabilitation and its outcome measures are feasible in LMICs [9,10]. Quality assurance procedures for pulmonary rehabilitation in the developed world, such as through quality standards and national audits, are now in place; helping to ensure the service patients receive is to a high standard. It is possible to support this development at an accelerated pace in LMICs through transfer of this quality assurance to local services. A common data set is being developed with four LMIC research and clinical institutes and with experts in pulmonary rehabilitation in the UK to help these new services be quality assured to a cutting edge standard.

## THE GLOBAL RECHARGE CORE DATA SET

The NIHR Global Health Research Group on Respiratory Rehabilitation (Global RECHARGE) brings together partners in Kyrgyzstan, India, Sri Lanka and Uganda, with the ultimate aim of restoring the health of people with chronic lung diseases through the development of new and effective pulmonary rehabilitation programmes. Major challenges to harmonising data across many nations, spanning several continents have included a variety of ethical, social and cultural contexts. Experts from India, Kyrgyzstan, Sri Lanka, Uganda and the United Kingdom (including clinicians, researchers, health psychologists, health economists, health care research methodologists and data scientists) were involved in establishing a common set of measures. Considering the breadth and depth of data collection and practicalities on the ground, consensus meetings informed by international guidelines [5,6] were used to develop a core set of variables to include in all pulmonary rehabilitation studies as a minimum. The data set is housed by a user-friendly web-based data capture tool called REDCap [11,12] hosted by the University of Leicester and administered by the NIHR Leicester Biomedical Research Centre’s Bioinformatics Team, as a central resource for LMIC groups interested in pulmonary rehabilitation. Data collection, data quality monitoring and data sharing are overseen by the consortium’s scientific committee which will also oversee requests to access the data from consortium partners and also external requests.

**Figure:**
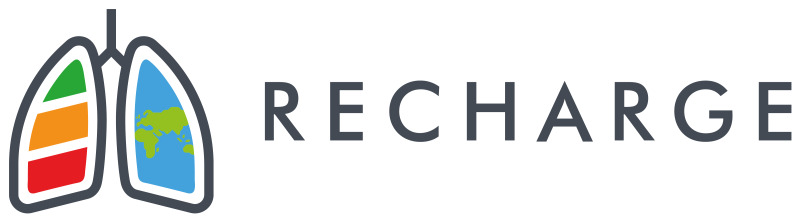
Photo: From Global RECHARGE, used with permission.

Standardising data collection with a pre-determined set of measurements, here referred to as the ‘Global RECHARGE Core Data set’, is proposed whereby collaborators will use common data collection tools and procedures. Through the use of a minimum set of common measures it becomes possible to describe and compare patient populations in LMICs. By using common outcomes, any change in delivery or settings can be compared to show what does and does not work. Benchmarking and quality improvement with continuous audit offer a potential to maximise benefits, reduce waste and improve patient outcomes. Large scale pooling of pulmonary rehabilitation data also offers unique research opportunities for now and into the future.

The Global RECHARGE Core Data set includes demographic variables and information about lung health, comorbidities and treatment, all captured at baseline before patients begin pulmonary rehabilitation. Demographics includes age, sex and ethnicity. Lung health measures include smoking status and biomass fuel exposure. Common comorbidities and treatments are also listed. Outcome measures, collected at baseline and at discharge assessment (after completing PR), comprise self-reported measures of health status, field-based tests of exercise capacity and functional status, and economic assessment. Health status included Medical Research Council (MRC) Dyspnoea Scale [13], COPD Assessment Test [14], Clinical COPD Questionnaire [15] and Hospital Anxiety and Depression Scale [16]. Physical measures are the Incremental Shuttle Walking Test (ISWT) [17] which is used as a field-based test of exercise capacity and the 5-times sit-to-stand [18] as a measure of functional status. The benefit of pulmonary rehabilitation may extend to supporting people’s return to being economically productive, such as through improvements in exercise capacity and symptoms. In the data set, economic impact is examined using the EQ5D5L [19] and Work Productivity and Activity Impairment questionnaire [20]. Pulmonary rehabilitation completion is recorded, including details about the frequency of pulmonary rehabilitation sessions, duration of the programme and attendance. The recording of adverse events allows for safety reporting, including the logging of all events and the specific cause of the event.

The visit schedule for the collection of data in accordance with the Global RECHARGE Core Data set measures is provided in **Table 1**. The Adverse Events data collection form has been placed within an unscheduled visit as this can be collected throughout the research process. Adverse events will be reported in real-time and there are no restrictions on the number of entries that can be made. Compacting the individual data sets as much as possible reduces the overall harmonisation task across the project and facilitates more efficient data entry and management.

**Table 1:** Visit schedule underpinning the Global RECHARGE Core Data set

Data collection	Baseline assessment	Discharge assessment	Unscheduled (during delivery and follow-up)
Demographics	x		
Lung health	x		
Comorbidities	x		
Treatment	x		
Health status	x	x	
Physical measures	x	x	
Economic impact	x	x	
Pulmonary rehabilitation completion		x	
Adverse events			x

## COLLABORATIONS AND DATA REQUESTS

RECHARGE will be publicised through social media (Twitter @Global_RECHARGE), on a website (https://www.globalrecharge.org.uk/), conference abstracts and peer-reviewed journal articles. For expressions of interest in collaborating, contributing research data to the database or for data access please contact the RECHARGE Scientific Committee by email at recharge@le.ac.uk.

We particularly encourage requests from health care providers and researchers from LMICs, including groups looking to strengthen their local research capacity. We welcome expressions of interest from those looking to set up pulmonary rehabilitation through to those already running a service. Support for interested parties will be provided by research and technical staff in Leicester, including developing data collection forms, data entry processes and standard operating procedures. Data from the Global RECHARGE Core Data set will be made available following the completion of this project and we are considering the best tools to use to make this database available to the wider community.

## ETHICS AND DATA GOVERNANCE

Data governance procedures for the Global RECHARGE Core Data set are set up in line with General Data Protection Regulation (EU GDPR) 2016/679 and the University of Leicester’s overall information governance framework, which sits under the wider umbrella of the University’s Research Code of Conduct (https://www2.le.ac.uk/offices/ias/dp, https://www2.le.ac.uk/services/research-data). For all collaborators to the data set, formal data sharing agreements will be established between Global RECHARGE and partners. Copies of local ethical approvals and study protocols will be required before access to the data set can be granted. Individual participant data will be allocated a unique non-identifiable study ID.

## ADDITIONAL CONSIDERATIONS

It is expected that partners will collect their own additional data set specific to their needs. Reasons for these additions may include compliance with their local data entry protocols and country- or disease-specific measures (eg, ethnicities which are appropriate for their region and screening data for Human Immunodeficiency Virus or Tuberculosis).

The application of the data set may be extended to other contexts in the future or act as a framework to support such developments. For example, many measures in the data set are clinically relevant to other chronic diseases, as well as infectious diseases including severe acute respiratory syndrome coronavirus 2 (SARS-CoV-2).

Education is a key component of pulmonary rehabilitation and there may be opportunities to harmonise or share best practices globally in this area. Some anticipated challenges in harmonising educational components are the need for tailored methods of delivery for different topics and populations (eg, lecture-based or discussion-based, written or verbal), the local availability of specialist health care staff, support services (eg, smoking cessation, psychological support), variations in medical provisions and some condition or location-specific issues (eg, stigma of infectious or post-infectious disease).

## FINAL THOUGHTS

It is particularly important for LMICs to adopt a robust data sharing and collection approach, enabling researchers globally to better address important health issues in a time- and cost-effective manner compared with isolated approaches to data acquisition. The value of data efficient and robust data collection, sharing and secondary analysis of publicly funded data sets is regarded highly by research funders. The wide adoption of the Global RECHARGE Core Data set will facilitate quality assurance of pulmonary rehabilitation programmes conducted in LMICs, provide opportunities to expand pulmonary rehabilitation services over time, de novo research opportunities offered by trans-national data and enhanced research capacity through driving data acquisition quality in partner organisations. We anticipate that this global minimum data set for pulmonary rehabilitation will help promote high impact research strategies, strengthen pulmonary rehabilitation advocacy in LMICs and provide greater returns on investments for all involved.
